# Broadening Horizons: Exploring mtDAMPs as a Mechanism and Potential Intervention Target in Cardiovascular Diseases

**DOI:** 10.14336/AD.2023.1130

**Published:** 2023-12-01

**Authors:** Yi Luan, Ying Luan, Yuxue Jiao, Hui Liu, Zhen Huang, Qi Feng, Jinyan Pei, Yang Yang, Kaidi Ren

**Affiliations:** ^1^Clinical Systems Biology Laboratories, The First Affiliated Hospital of Zhengzhou University, Zhengzhou, China.; ^2^State Key Laboratory for Artificial Microstructures and Mesoscopic Physics, School of Physics, Peking University, Beijing, China.; ^3^School of Laboratory Medicine, Xinxiang Medical University, Xinxiang, China.; ^4^Department ofIntegrated Traditional and Western Nephrology, The First Affiliated Hospital of Zhengzhou University, Zhengzhou, China.; ^5^Quality Management Department, The Third People’s Hospital of Henan Provine, Zhengzhou, China.; ^6^Department of Pharmacy, the First Affiliated Hospital of Zhengzhou University, Zhengzhou, China.; ^7^Henan Key Laboratory of Precision Clinical Pharmacy, Zhengzhou University, Zhengzhou, China.

**Keywords:** Inflammation, damage-associated molecular patterns (DAMPs), cardiovascular diseases (CVDs), mitochondrial, immune response

## Abstract

Cardiovascular diseases (CVDs) have been recognized as the leading cause of premature mortality and morbidity worldwide despite significant advances in therapeutics. Inflammation is a key factor in CVD progression. Once stress stimulates cells, they release cellular compartments known as damage-associated molecular patterns (DAMPs). Mitochondria can release mitochondrial DAMPs (mtDAMPs) to initiate an immune response when stimulated with cellular stress. Investigating the molecular mechanisms underlying the DAMPs that regulate CVD progression is crucial for improving CVDs. Herein, we discuss the composition and mechanism of DAMPs, the significance of mtDAMPs in cellular inflammation, the presence of mtDAMPs in different types of cells, and the main signaling pathways associated with mtDAMPs. Based on this, we determined the role of DAMPs in CVDs and the effects of mtDAMP intervention on CVD progression. By offering a fresh perspective and comprehensive insights into the molecular mechanisms of DAMPs, this review seeks to provide important theoretical foundations for developing drugs targeting CVDs.

## 1.Introduction

Cardiovascular diseases (CVDs) are a ubiquitous cause of morbidity and one of the leading contributors to mortality globally despite significant advances in therapeutics [1, 2]. Therefore, it is imperative to develop novel options to combat CVDs that are commonly associated with inflammation. Inflammation, a reparative response to tissue injury, is critical for preventing infection and improving CVDs [3]. Although limited cardiovascular inflammation benefits from cardiac injury, excessive chronic inflammation can induce serious tissue damage if not terminated after tissue repair. Previous studies have suggested that inflammation is crucial for CVD progression, including myocardial infarction (MI), atherosclerosis, and hypertrophic heart failure [4].

Cardiovascular inflammation is normally accompanied by increased levels of cytokines, including interleukin-1β (IL-1β), IL-6, and tumor necrosis factor (TNF), and decreased levels of anti-inflammatory cytokines, including IL-10 and transforming growth factor-beta (TGF-β), which are produced by the immune cells [5, 6]. In addition to inflammatory immune cells, cardiovascular cells, including cardiomyocytes, endothelial cells, and cardiac fibroblasts, can produce inflammatory cytokines in response to various stimuli.

Despite its role in cardiovascular inflammation, the heart can reduce inflammation through inherent and endogenous mechanisms. Cardiac inflammation and injury can be mitigated by the heart through two mechanisms: regulating the levels of proinflammatory mediators or augmenting the levels of anti-inflammatory mediators [7].

**Figure 1.**
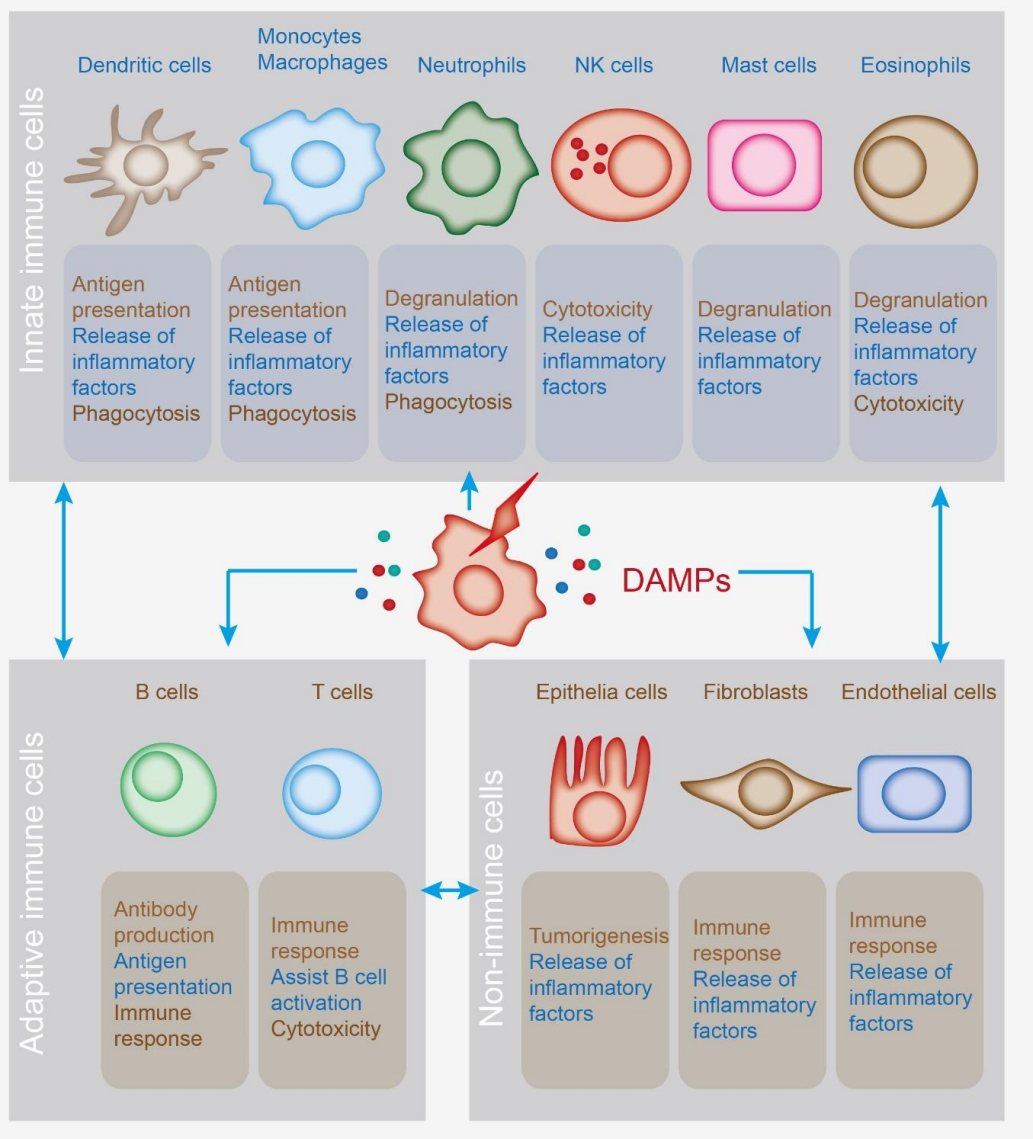
**Several cell types are involved in DAMP-induced inflammation**. Dendritic cells (DCs), neutrophils, monocytes/macrophages, natural killer (NK) cells, mast cells, and eosinophils can release multiple proinflammatory mediators when stimulated by DAMPs, leading to the recruitment of inflammatory cells. Additionally, nonimmune cells, encompassing epithelial cells, endothelial cells, and fibroblasts, produce various immune receptors and can be activated by DAMPs.

## 2.Inflammation in CVDs

Inflammation plays an important role in CVD progression. Inflammation involves several finely regulated steps. First, exogenous or endogenous stimuli trigger an inflammatory response. Second, downstream pattern recognition receptor (PRR) signaling is activated, which induces elevated expression of inflammatory cytokines, chemokines, and vasoactive amines. Subsequently, these factors are released outside the cells. Finally, immune cells, including neutrophils, are recruited to remove microorganisms by accumulating proteases, ROS, or radical nitrogen species [8]. Macrophages also participate in the removal of ligands or damaged tissues. In terms of heart-related diseases, cardiac inflammation can be divided into two pathological aspects: inflammatory cardiomyopathies, such as myocarditis, and cardiac inflammation [9]. Myocarditis induced by viruses or protozoa is associated with a massive inflammatory response and commonly results in heart dysfunction. Cardiac inflammation refers to noninfectious inflammation, also called “sterile inflammation,” which is often induced by myocardial damage as a secondary response.

As previously reported, cardiac inflammation drives the pathogenesis of heart failure [10]. Inflammatory cytokine levels are enriched in patients with heart failure. Elevated levels of cytokines and receptors act as independent risk factors for mortality in patients with advanced heart failure patients [11]. Serum levels of TNF-α are positively correlated with the severity of heart failure. This may be attributed to the downregulated levels of Ca^2+^-cycling-associated genes, including sarcoplasmic reticulum Ca^2+^ ATPase (SERCA2), mediated by elevated TNF-α and IL-1β by activating NF-κB. Genes associated with altered Ca^2+^ cycling induce weakened contractility by altering intercellular Ca^2+^ homeostasis in adult cardiomyocytes. Disturbed Ca^2+^ homeostasis induced by inflammation in cardiomyocytes is potentially associated with cardiac remodeling, constituting a vicious cycle [12]. Moreover, TNF-α and IL-1β contribute to cardiac hypertrophy, forming another risk factor for heart failure. Additionally, IL-6 has been found to induce cardiomyocyte stiffness by inhibiting titin phosphorylation [13].

In the heart, cytokines are produced by several cell types, including cardiomyocytes, cardiac fibroblasts, endothelial cells, and resident macrophages (Fig. 1) [14]. Furthermore, the released cytokines can recruit immune cells into the heart, including neutrophils and macrophages, producing cytokines and chemokines under pathological conditions (Fig. 1). These factors may induce consecutive chronic inflammation in the heart. However, the mechanisms underlying the initiation and maintenance of chronic inflammation remain unclear.

TGF-β modulates the transdifferentiation of fibroblasts into active myofibroblasts [15]. Myofibroblasts present higher levels of collagen and inflammatory cytokines than quiescent fibroblasts do. Activated myofibroblasts induce cardiac hypertrophy and dysfunction by producing pro-hypertrophic factors, including angiotensin II (Ang II), TGF-β1, and fibroblast growth factor (FGF) [16]. Myofibroblasts can also induce gelatinase expression, making the microvasculature more permeable to subsequent immune cell infiltration into the heart and regulating macrophage polarity. Cardiac endothelial cells are key producers of IL-1β, one of the end products of NLRP3 inflammasome activation.

In addition to endogenous cardiac cells, infiltrating immune cells modulate cardiac inflammation (Fig. 1) [17]. Neutrophils can generate large amounts of ROS, which act as host defenses and induce tissue damage. Macrophages can also benefit from cardiac inflammation by polarizing M2 macrophages and alleviating inflammation through phagocytosis.

### 2.1 Inflammation in MI

MI occurs when the coronary arteries are blocked, leading to the death of cardiac muscle cells [18]. Patients with acute MI have increased plasma TNF-α, granulocyte colony-stimulating factor (GCSF), granulocyte-macrophage colony-stimulating factor (GM-CSF), macrophage colony-stimulating factor (MCSF), interferon-alpha (IFNα), and interferon-beta (IFNβ) levels. The release of proinflammatory cytokines during MI is driven by neutrophils, macrophages, and mast cells [19].

Neutrophil infiltration increases within 24 h of MI, accompanied by IL-1β release through the S100A8/9-TLR4-inflammasome axis, as confirmed by several studies [20, 21]. Subsequently, IL-1β interacts with its receptor on hematopoietic stem and progenitor cells, promoting granulopoiesis while impairing cardiac function [22]. Long-term S100A9 blockade negatively affects cardiac recovery and counterbalances the beneficial effects of short-term therapy [23]. Mast cells also induce local inflammation during MI through the release of histamine and their role in cytokine production [24]. Macrophages play a crucial role in regulating inflammation during MI by releasing both inflammatory and anti-inflammatory cytokines [25]. For example, during the first 1-3 days after MI, macrophages primarily release proinflammatory cytokines, including GM-CSF, IFN-γ, TNF-α, and IL-1β. Conversely, they produce anti-inflammatory mediators, including IL-10, vascular endothelial growth factor (VEGF), and TGF-β at 5-10 days post-MI.

Macrophages are polarized into different states by cytokines [14]. Both IFNγ and GM-CSF are proinflammatory mediators, while IL-4, IL-10, IL-13, and TGF-β act as anti-inflammatory cytokines that promote macrophage polarization. Remote monocytes also play a role in cardiac inflammation following MI [26]. Lymphocyte antigen 6 complex (Ly6C) high monocytes transform into inflammatory macrophages through the C-C chemokine receptor type 2 pathway, while Ly6C low monocytes differentiate into anti-inflammatory macrophages through the CX3CR1 pathway in response to acute cardiac stress [26]. Moreover, IL-10 has been found to ameliorate cardiac inflammation and improve cardiac function by polarizing macrophages toward an anti-inflammatory phenotype.

Furthermore, the infiltration of T cells, including CD8^+^ and CD4^+^ T cells, is increased in ischemic and hypertrophic hearts. The depletion of CD4^+^ T cells alleviates heart failure following MI [27]. However, transferring spleen-derived CD4^+^ T cells to healthy mice leads to fibrosis and hypertrophy [28]. Briefly, the occurrence of varied immune cell infiltration following MI initiates cardiac remodeling, suggesting that therapies targeting the suppression of immune cell infiltration may provide beneficial results in managing ischemic heart injury.

### 2.2 Inflammation in cardiac hypertrophy

Cardiac hypertrophy is characterized by enlarged cardiomyocytes and fibrosis, eventually leading to heart failure [29]. Recent evidence suggests that immune cells and inflammatory signaling are crucial in nonischemic heart injuries, including cardiac hypertrophy [30]. The recruitment of leukocytes to the myocardium drives the hypertrophic growth of the heart. Furthermore, reduced recruitment of monocytes improves cardiac hypertrophy in response to chronic stress [31]. For instance, monocyte chemoattractant protein-1 (MCP1) depletion suppresses chronic hypertrophic cardiac remodeling [32].

Additionally, the interaction between IFNγ^+^ T cells (Th1) and cardiac fibroblasts induces TGF-β expression, indicating the coordination of T cells and cytokines in cardiac fibrosis [33]. As previously speculated, ablation of TCR-α alleviates cardiac fibrosis and heart failure [34]. Microbiomes can also modulate cardiac hypertrophy through T-cell-dependent metabolites [35]. The levels of proinflammatory cytokines increase in patients with dilated cardiomyopathy [36]. Previous studies have disclosed that IL-1β and TNF-α levels are elevated in cardiomyocytes treated with isoproterenol (ISO), whereas IL-10 treatment significantly reduces the induction of these cytokines in a STAT3-dependent manner. Immune cell infiltration and inflammatory cytokines are important regulators in hypertrophic cardiac remodeling. Moreover, it is worth noting that anti-inflammatory therapy has the potential to mitigate cardiac injury.

### 2.3 Inflammation in the aging heart

Inflammaging is a chronic, mild, and sterile inflammation that occurs in older individuals. This condition is associated with various factors, including mitochondrial dysfunction, inflammation, immune cell senescence, and genomic stability [37]. Ultimately, inflammaging can lead to age-related heart diseases [38]. Aging triggers the release of different substances, including metabolites, mitochondrial DNA (mtDNA), and cytokines, which induce inflammation. Inflammaging has implications for several disorders, including atherosclerosis, hypertension, and hypertrophic remodeling [39]. Numerous immune cells, including macrophages, dendritic cells (DCs), neutrophils, B cells, and T cells, result in inflammaging development. For instance, as individuals age, the number of resident anti-inflammatory macrophages (F4/80^+^CD206^+^) in the heart declines, while the population of inflammatory macrophages (F4/80^+^ CD206^-^) increases [40].

Several proteins are altered during inflammaging progression. Elevated MCP1, an important chemokine for macrophage recruitment, was observed in the left ventricle of aged mice [41]. Additionally, the level of matrix metallopeptidase 9 (MMP9), which is involved in cardiac hypertrophy and fibrosis, is enriched. Therefore, MCP1 and MMP9 are recognized as biomarkers of cardiac aging, and further exploration is necessary to determine their specific roles in human cardiac aging [42]. In aging mice, the levels of inflammatory cytokines in the plasma increase, along with the levels of MCP1, MCP2, and macrophage inflammatory protein 2 (MIP2), which are related to the left ventricular end-diastolic dimensions. MMP9 ablation increases the number of anti-inflammatory macrophages and restores cardiac function in the hearts of aged mice [43].

Interestingly, administration of atorvastatin, a lipid-reducing agent, reduces MMP9 levels and restores the left ventricle, cardiomyocyte size, and collagen deposition in aged rats [44]. Conversely, overexpression of MMP9 in macrophages exacerbates inflammation, ventricular hypertrophy, and cardiac fibrosis in aged mice [45]. These studies highlight the function of MMP9 in the pathogenesis of cardiac aging.

Genetic ablation of MMP28, which modulates macrophage polarization toward an anti-inflammatory phenotype, increases MMP9 levels and worsens inflammation in the aged ventricle [46]. The levels of inflammatory genes decrease, while the levels of fibrosis genes increase in the macrophages of aged mice, indicating that macrophages are essential for inflammaging.

T cells have been thoroughly explored as immune cells in cardiac inflammaging [47]. The CD4^+^ T cells from elderly individuals exhibit an inflammatory Th17 phenotype, characterized by mitochondrial dysfunction and abnormal autophagy upon *in vitro* activation [48]. Metformin therapy has been shown to convert the Th17 phenotype to a CD4^+^ T cell phenotype in younger individuals, restoring normal mitochondrial function and autophagy processes [49]. Furthermore, suppressing ROS production suppresses the Th17 phenotype, suggesting a mutual impact of mitochondrial stress, oxidative stress, and Th17 in inflammaging [50].

## 3.Inflammation sensors and signaling pathways

**Figure 2.**
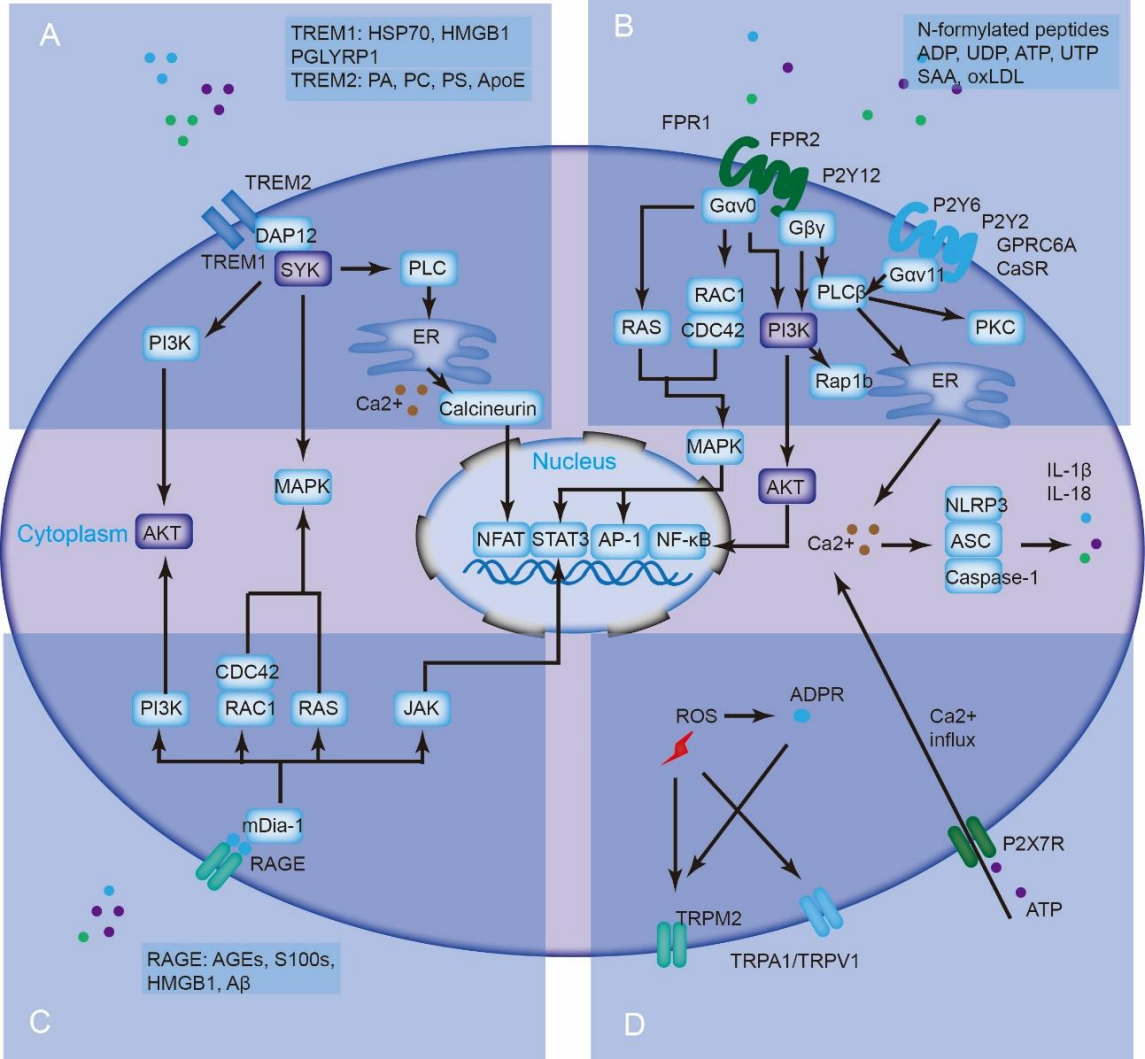
**Multiple transmembrane proteins can be receptors for endogenous DAMPs, inducing multiple signaling pathways and promoting the recruitment of various immune cells**. These receptors include TREMs, GPCRs, RAGE, and TRP and P2X7R channels.

As widely acknowledged, pathogen-associated molecular patterns (PAMPs) and DAMPs can trigger an immune response by activating classical PRRs, including toll-like receptors (TLRs), as well as other germ-line-encoded receptors, such as NOD-like receptors (NLRs), retinoic acid-inducible gene I (RIG-I)-like receptors (RLRs), C-type lectin receptors (CLRs), and multiple intracellular DNA sensors [51]. In addition, multiple transmembrane proteins can act as receptors for endogenous DAMPs, inducing several signaling pathways and promoting the recruitment of various immune cells. These receptors include triggering receptors expressed on myeloid cells (TREMs), G-protein-coupled receptors (GPCRs), advanced glycation end products (RAGE), transient receptor potential (TRP), and P2X7 receptor (P2X7R) channels (Fig. 2).

TREM1 and TREM2 are key receptors of the TREM family and can be recognized by multiple proteins and lipids [52]. Specifically, TREM1 can be activated by four endogenous molecules: high-mobility group box 1 (HMGB1), heat shock protein 70 (HSP70), peptidoglycan recognition protein 1 (PGLYRP1), and extracellular actin (Fig. 2). TREM2 can be recognized by numerous lipids such as phosphatidic acid (PA), phosphatidylcholine (PC), phosphatidylserine (PS), lipoproteins apolipoprotein E (APOE), and low-density lipoprotein (LDL). Following activation, TREM1, and TREM2 can bind to and phosphorylate the adaptor protein DNAX-activating protein of 12 kDa (DAP12), inducing SYK activation and subsequent downstream signaling pathways, including Ca^2+^ mobilization, PI3K, and mitogen-activated protein kinase (MAPK) activation [53].

GPCRs can bind to endogenous DAMPs to induce sterile inflammation [51]. These GPCRs include N-formyl peptide receptors (FPRs), which can sense both N-formylated and non-formylated peptides, P2Y receptors (P2YRs) for extracellular nucleotides, and calcium-sensing receptor (CaSR) and GPCR family C group 6 member A (GPRC6A) for extracellular Ca^2+^. These GPCRs couple heterotrimeric G proteins with different subtypes of Gα subunits (Gαs, Gαi/o, Gαq/11, and Gα12/13) and Gβγ subunits, mediating the migration and recruitment of immune cells (Fig. 2) [54]. The activation of FPR1 and FPR2 induces the activation of multiple GTPases of the RAS superfamily (RAS, RAC, and CDC42) by coupling with Gαi/o and the subsequent activation of the MAPK pathway [55]. Similarly, P2Y12 initiates the activation of RAP1b and AKT via the PI3K pathway, accompanied by Gαi/o coupling. Furthermore, Gαi/o blocks the adenylate cyclase (ADCY)-cyclic AMP (cAMP) signaling pathway [56]. P2Y2 and P2Y6 activate downstream signaling by coupling with Gαq/11. Gαq/11 induces phospholipase Cβ (PLCβ), which mobilizes Ca^2+^ release or induces protein kinase C (PKC) activation [57]. CaSR and GPRC6A coupled with Gαq/11 promote NLRP3 inflammasome activation by releasing Ca^2+^ from the ER. In addition to these Gα subunits, GPCRs can be coupled with Gβγ subunits, promoting both activation of the PI3K-AKT pathway and PLCβ.

RAGE can bind to endogenous ligands, including advanced glycation end products (AGEs), HMGB1, S100s, and β-amyloid (Aβ), initiating a proinflammatory response (Fig. 2) [51]. RAGE signaling is complicated, and its recognition is also relevant to cell type. Mammalian diaphanous 1 (mDia-1) is a downstream adapter protein of RAGE. Multiple signaling pathways, including the PI3K-AKT, MAPK, and JAK-STAT pathways, can be activated after RAGE activation [58]. Among these pathways, several transcription factors, including NF-κB, activator protein 1 (AP-1), and STAT3, promote the expression of diverse inflammatory genes and genes related to cell migration, proliferation, and apoptosis.

TRP channels (TRPM2, TRPA1, and TRPV1) can be activated by ROS-dependent or independent intracellular ADP-ribose (ADPR) (Fig. 2) [59]. These nonselective calcium channels enable free Ca^2+^ influx. The influx of calcium can facilitate the initiation of several biological processes, including MAPK pathways and nuclear factor-related proteins related to activated T cells (NFAT) [60]. Moreover, calcium influx mediated by TRPM2 combines ROS production with NLRP3 inflammasome activation. ATP stimulation via P2X7R induces a nonselective influx of Ca^2+^ and K^+^ efflux [61]. Furthermore, P2X7R-mediated influx activates calcineurin, inducing NFAT dephosphorylation and translocation to the nucleus. K^+^ efflux is an upstream event that activates NLRP3 inflammasome [62]. Furthermore, the elevation of intracellular Ca^2+^ serves as a stimulatory factor within the NLRP3 inflammasome.

## 4.Role of mitochondrial dysfunction in CVDs

CVD pathogenesis is complicated and involves numerous risk factors and pathological mechanisms. Within cells, various abnormalities, such as metabolic defects, excessive ROS production, and energy deficits, result in CVD progression [63]. Mitochondrial dysfunction is recognized as a key factor in cellular perturbations.

Cardiac pathologies are often accompanied by structural abnormalities in mitochondria, including giant mitochondrial formation. During heart ischemia, fragmented mitochondria are induced by upregulation of dynamin-related protein 1 (Dnp1) [63]. This protein forms a pro-apoptotic complex with Mfn2 and Bax, leading to mitochondrial fragmentation and cardiomyocyte apoptosis [64]. Considering this information, inhibiting Dnp1 has been shown to inhibit cardiac mitochondrial fragmentation and subsequent apoptosis.

Mitochondrial dysfunction is also essential for developing atherosclerosis as it contributes to oxidative stress, which is a critical factor. Excessive ROS can induce endothelial cell senescence, leading to apoptosis and atherosclerotic plaque formation [65]. Furthermore, dysfunctional mitochondria can cause the translocation of various molecules from the mitochondria to other parts of the cell, including the cytosol and extracellular space, thereby triggering an immune response [66]. The cellular response to pathogen invasion involves the activation of defense mechanisms wherein cells secrete inflammatory cytokines and chemokines, thereby stimulating both the adaptive immune system and phagocytosis. All these studies indicate the influence of mitochondrial dysfunction on CVDs.

## 5.Effects of mitochondria on inflammatory response

An increasing number of studies suggest a potential connection between mitochondria and inflammation. Numerous mitochondrial constituents and metabolic products can serve as DAMPs and promote inflammation when released into the cytosol or extracellular milieu [67]. For instance, fumarate (an intermediate in metabolic processes in mitochondria) triggers the activation of specific inflammation-related pathways [68]. In addition, the mitochondria can directly trigger inflammatory responses. Mitochondria activate mitochondrial antiviral signaling and NLRP3 inflammasome. In immune cells, mitochondria are located adjacent to the endoplasmic reticulum (ER); consequently, mitochondria and ER-tethered signaling can regulate immune cell metabolism. Mitochondrial machinery, including mitochondrial dynamics, mtDNA, mitophagy, and mtROS, is crucial for immune functions. Mitochondrial stress leads to the release of DAMPs that can activate innate immunity. Mitophagy can also mitigate inflammation [69]. Parkin and PINK1 have been proposed to prevent inflammation and neurodegeneration by clearing damaged mitochondria to prevent increases in cytosolic and circulating mtDNA levels [69]. In short, the link between mitochondria and inflammation is greater than previously understood, and understanding their interactions can help fully understand the diseases that result.

When cells are subjected to mechanical stress, they release cellular compartments outside the cell, known as DAMPs. DAMPs can also be recognized by receptors, such as TLRs and NOD, thereby inducing additional immune response activation [51]. Mitochondria have recently been recognized as a significant source of DAMPs. During cell death, the mitochondria release mtDAMPs to initiate an immune response when stimulated by cellular stress.

**Figure 3.**
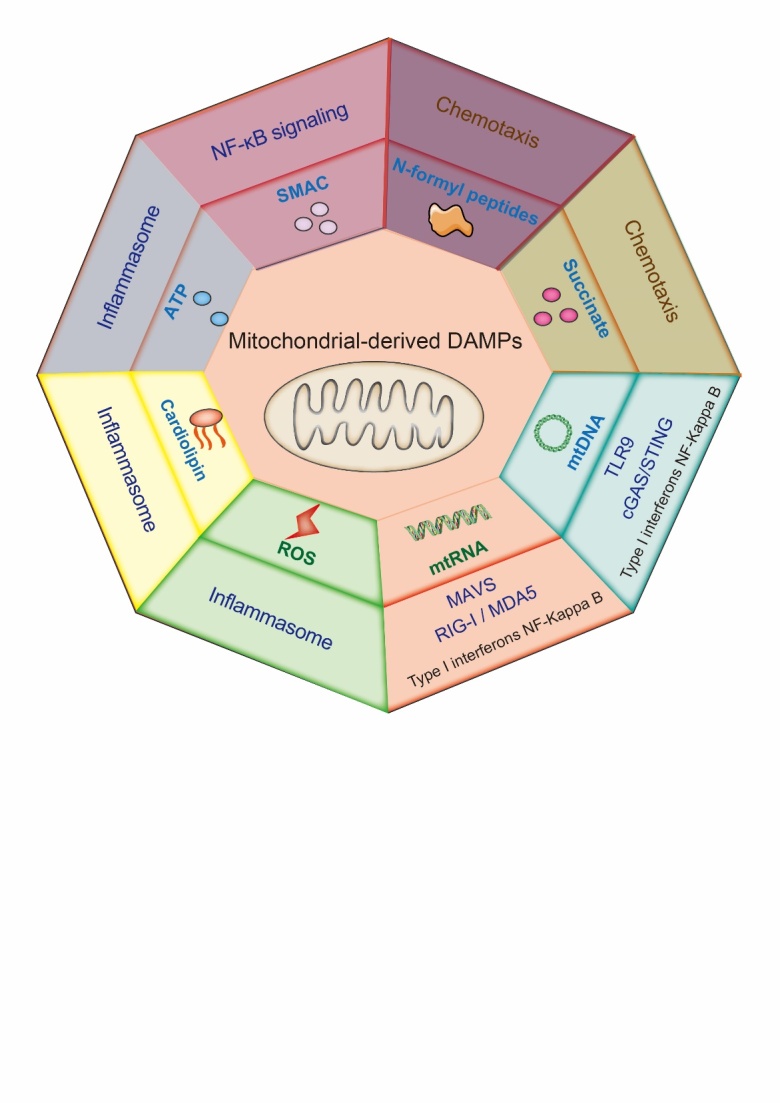
**Outline of mitochondrial-derived DAMPs**. Mitochondria can release DAMPs that can induce an immune response, including NFPs, succinate, mtDNA, mtRNA, ROS, cardiolipin, ATP, and the second mitochondrial-derived activator of caspase (SMAC). These DAMPs activate different immune pathways.

## 6.mtDAMPs

Several mtDAMPs can be released from mitochondria under mitochondrial stress or damage, including N-formyl peptides (NFPs), mtDNA, cardiolipin, and ROS, as well as metabolites, including ATP and succinate (Fig. 3) [70].

NFPs are mainly detected in bacteria where formyl-modified methionine participates in protein synthesis [70]. These NFPs act as chemoattractants for host phagocytes and are recognized by their receptors. Mitochondrial methionine formylation is essential for transcriptional initiation of mtDNA-derived mRNA [71]. Succinate, a mitochondrial metabolite, initiates the immune response in DCs [72].

Among these mtDAMPs, mtDNA has remained the most well-defined DAMP in the past few years, especially in mitochondrial apoptosis. A notable similarity was observed between mtDNA and bacterial DNA in terms of their detection by TLR9. Cytosolic DNA can also be recognized by 2'3'-cyclic GMP-AMP (cGAMP) synthase (cGAS), which induces an interferon type I response by the stimulator of interferon genes (STING) (Fig. 3) [73]. Cardiolipin phospholipid is the third mtDAMP shared with bacteria, and its stimulation can induce mitophagy [74]. Cardiolipin also induces mitochondrial apoptosis by promoting BAX pore formation. However, the degree of cardiolipin required for apoptosis is highly variable. Cardiolipin can also induce the NOD-like receptor family pyrin domain containing 3 (NLRP3) inflammasome in the presence of PAMPs or antibiotics (Fig. 3) [75]. Both mtDNA and mitochondrial ROS can also promote NLRP3 inflammasome activation.

## 7.mtDAMPs-induced signaling pathways

Upon release, DAMPs activate caspase-1 and induce the secretion of proinflammatory cytokines [76]. Notably, mtDNA has been considered a subtype of DAMPs and is associated with neurodegeneration. It initiates various inflammatory responses by binding to three different sensors: Toll-like receptor (TLR), NOD-like receptor (NLR), and cyclic GMP-AMP synthase-stimulator of interferon genes (cGAS-STING) [77].

### 7.1 TLR pathway

The binding of DAMP to neutrophils activates the TLR pathway, followed by inflammation organization of inflammation via the NF-κB pathway [78]. TLR9, an endosomal TLR that binds bacterial and viral DNAs, can subsequently activate downstream pathways via MyD88 [79]. Activation of the NF-κB pathway facilitates the transcriptional elevation of the proinflammatory cytokines IL-6 and pro-IL-1β, as well as NLRP3 and type 1 IFN. TLR9 activation is the initial step of NLRP3 inflammasome activation [80].

### 7.2 NLR pathway

**Figure 4.**
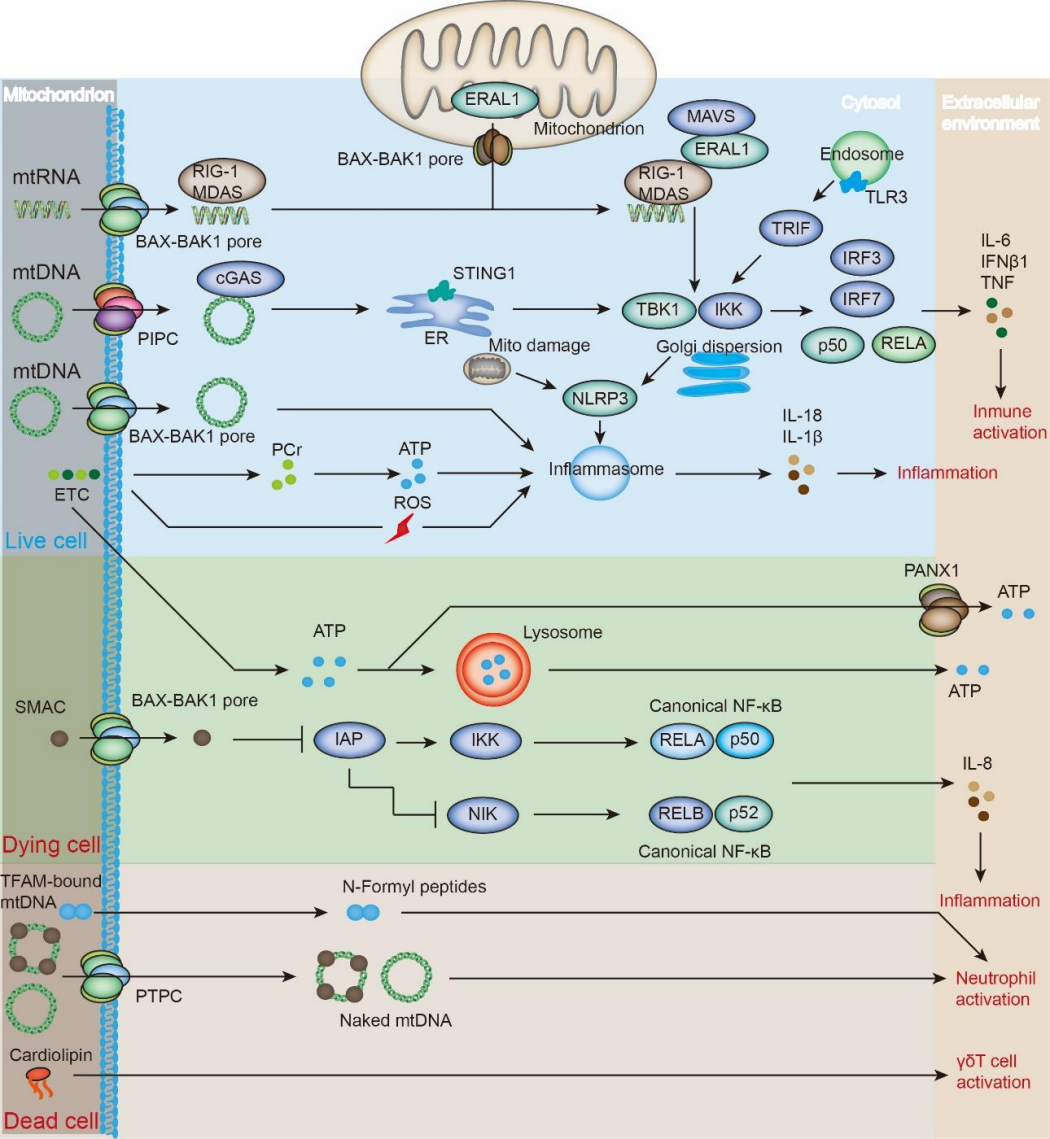
**Mitochondrial DAMP signaling mechanisms**. mtDNA activates cGAS-STING1 signaling and produces cytokines, including IFNβ1, IL-6, and TNF. mtRNA activates the RIG-I-MDA5 pathway. Both mtDNA and ROS induce IL-1β and IL-18 secretion, subsequently promoting inflammasome signaling. ATP can be recognized by the purinergic receptors P2RX7 and P2RY2. SMAC release not only induces caspase activation but also rewires NF-κB signaling. Extracellular NFPs potently activate neutrophils by binding to their receptors. Extracellular cardiolipin not only induces the elevation of MHC class I-like molecule CD1d on APCs but also binds to CD1d on the APC surface, contributing to the activation of cardiolipin-specific T cells with unconventional γδ T-cell receptor expression.

Another DAMP-activated pathway involves the NLRP3 inflammasome, which is composed of a sensor (NLRP3), an adaptor (ASC; also known as PYCARD), and an effector (caspase 1) [81]. The NLRP3 recognizes multiple danger signals, including toxins, viruses, and crystalline cholesterol. The mtROS mediates NLRP3 activation, which may be attributed to its oxidizing function in mtDNA [82]. mtROS leads to increased translocation of oxidized mtDNA, which activates the NLRP3 inflammasome (Fig. 4) [83]. Upon activation, NLRP3 co-localizes with ASC on the endoplasmic reticulum (ER)-mitochondrial membrane and further induces caspase-1 activation. Subsequently, caspase-1 cleaves and activates IL-1β and IL-18. Genetic ablation of *NLRP3* and *caspase-1* reduces mtDNA release [84]. Moreover, non-oxidized mtDNA induces IL-1β production by activating other inflammasomes, including those absent from melanoma 2 (AIM2).

### 7.3 cGAS-STING pathway

Another major sensor that activates the innate immune system is the cGAS-STING pathway. STING, an ER-localized protein, can induce IFN response by directly binding to dsDNA or cyclic dinucleotides. Furthermore, cGAS can act as a DNA sensor [85]. The binding of cGAS to mtDNA triggers the recruitment of stimulators of STING and facilitates the phosphorylation of the transcription factor IRF3 through the involvement of TANK-binding kinase (TBK) (Fig. 4) [86]. Activated IRF3 further promotes the generation of type I and III IFNs and IFN downstream proteins.

Sustained inflammatory initiation can recruit circulating immune cells, leading to a systemic inflammatory response through mtDNA-induced inflammatory response. The released components of inflammatory cells, including cytokines, chemokines, nitric oxide (NO), and ROS, may further exacerbate mitochondrial damage, forming a vicious cycle [87]. The administration of mtDNA through systemic injection upregulated IFN-related gene expression in the spleens of wild-type mice. Conversely, mtDNA injection in animals lacking the STING protein did not elicit the same response. Similarly, mitochondrial RNA (mtRNA) can induce an immune response through RIG-I, melanoma differentiation-associated protein 5 (MDA5), and mitochondrial antiviral signaling protein (MAVS). This pathway is favored by the Era-like 12S mitochondrial rRNA chaperone 1 (ERAL1), which promotes MAVS stabilization at the mitochondrial surface (Fig. 4).

### 7.4 Other signaling pathways

In addition to the major pathways (TLR and NLR pathways) mentioned above, several other mtDAMPs, namely the second mitochondria-derived activator of caspase (SMAC), NFPs, cardiolipin, and cytochrome C, are implicated in the inflammatory response [66].

Pro-apoptotic and proinflammatory signaling pathways induce SMAC release via the inhibitor of apoptosis (IAP) family (Fig. 4). IAP inhibition by SMAC rapidly induces NF-κB signaling by stabilizing mitogen-activated protein kinase 14 (MAP3K14, also known as NIK), a process also coordinated by Bax-Bak1 oligomers [88]. Consequently, ablation of IAP-encoded Birc2 and Birc3 leads to abnormal cell death and inflammation, which can be restored by Caspase 8 depletion plus NIK inhibition [89]. Furthermore, SMAC mimetics have anticancer effects that are partially attributed to the activation of the antitumor immune response upon macrophage repolarization. Notably, SMAC can directly modify the immune response by inducing the transformation of CD4^+^ T cells from a Th17 to Th2-type phenotype [90]. Therefore, mtDNA typically induces various inflammatory responses that are dependent on TBK1, whereas SMAC-triggered inflammation is probably mainly dependent on NF-κB signaling.

NFPs and cardiolipin are generally located in the mitochondrial matrix and inner mitochondrial membrane. Extracellular NFPs potently activate neutrophils by binding to their receptors [91]. Extracellular cardiolipin induces elevation of the MHC class I-like molecule CD1d in antigen-presenting cells (APCs). It binds to CD1d on the APC surface, contributing to the activation of cardiolipin-specific T cells with unconventional γδ T cell receptor expression (Fig. 4).

From a precise standpoint, it can be noted that ATP and heme are not conventionally regarded as integral constituents of mitochondria. However, it is worth mentioning that these components can indeed be generated within the mitochondria and modulate the immune response when released into the extracellular microenvironment. ATP released by cancer cells can induce immunogenic cell death by recruiting APC precursors to the TME in a P2RY2-dependent manner, resulting in inflammation activation and IL-1β production to maintain immunosurveillance (Fig. 4) [92]. Extracellular heme has been proven to be involved in multiple immunoregulatory processes, including the activation of endothelial cells and microglia, NLRP3-dependent caspase-1 activation-induced IL-1β release, and NLRP3-independent caspase 4 and caspase 5 activation. Numerous mitochondrial components can induce immune responses via different mechanisms.

## 8.mtDNA and cardiac inflammation

Although circulating mtDNA has been observed in the plasma and serum of several human diseases, there is limited evidence that directly reveals the roles of mtDNA in cardiac inflammation. Circulating mtDNA is elevated due to aging and is positively correlated with the levels of proinflammatory cytokines. Moreover, circulating mtDNA can induce cytokine generation in monocytes, and this inflammatory response is potentially involved in age-related CVDs, including ischemic heart diseases, heart failure, and atherosclerosis. Notably, the function of mtDNA-mediated inflammation in cardiac diseases has been verified in genetically engineered mouse models [93, 94].

Besides mtDNA sensors, molecules involved in mtDNA regulation may also participate in cardiac inflammation; however, their physiological roles remain inadequately defined. For example, overexpression of mtDNA-binding protein transcription factors (TFAM) protected cardiac pathological models. Conversely, the role of mtDNA in cardiac inflammation in these models remains unclear [95]. Ablation of cyclophilin D (CypD) inhibits mitochondrial permeability transition (MPT), which is responsible for mtDNA release and protects against ischemia/reperfusion injury [96]. The mechanism remains elusive, except for the inhibition of necrotic cell death. The role of NFPs, a form of mtDAMP, in CVD pathogenesis remains unknown. Moreover, the function of cardiolipin in CVD remains unexplored, although its effect on mitochondrial function and morphology in the heart has been discussed.

## 9.mtDAMPs and CVD

### 9.1 Ischemic heart diseases

**Figure 5.**
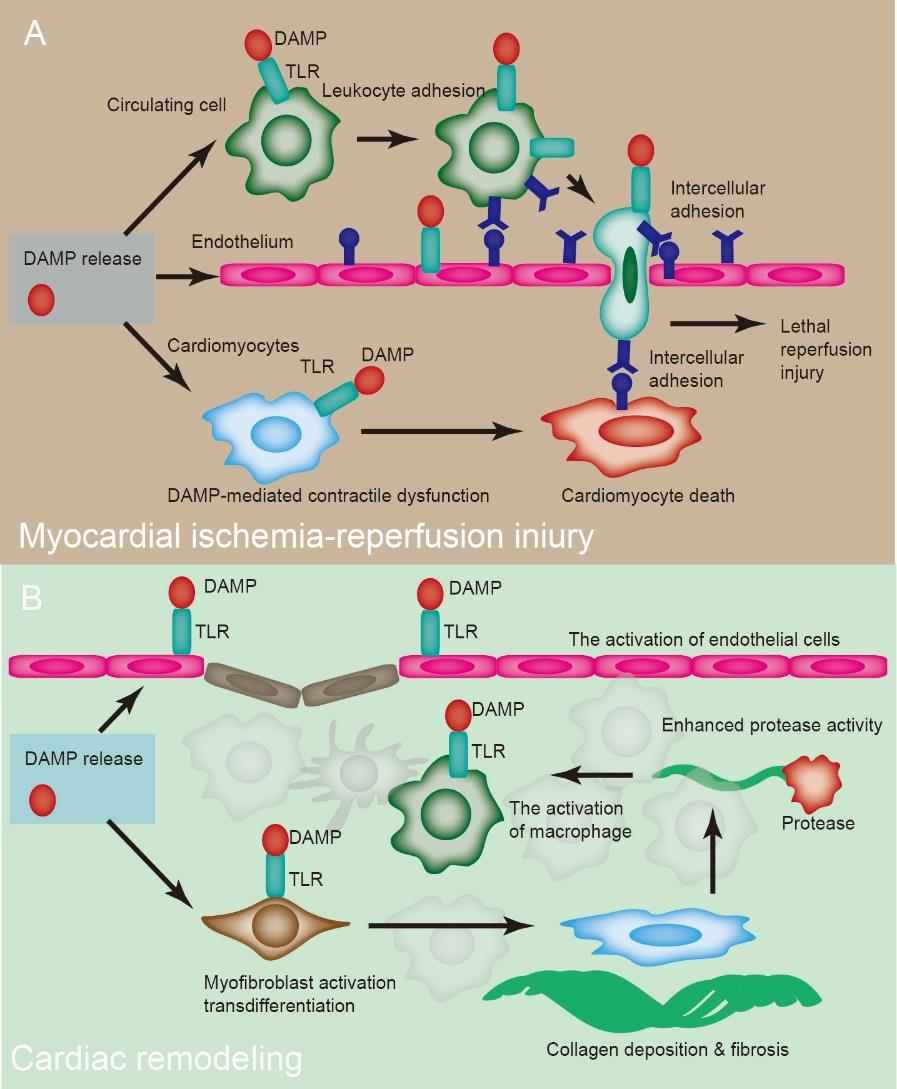
**DAMPs in cardiac remodeling and ischemia-reperfusion injury**. DAMPs, released after ischemia-reperfusion injury, induce TLR activation, resulting in deterioration of contractility and apoptosis. Reperfusion first induces endothelial dysfunction, followed by vasoconstriction and abnormal blood flow due to leukocyte adhesion, which can directly induce the death of cardiac and endothelial cells. Components released by dead cardiomyocytes and matrix degradation can recruit macrophages and fibroblasts.

DAMPs, released after ischemia-reperfusion injury, induce TLR activation, resulting in the deterioration of contractility and apoptosis. Reperfusion first induces endothelial dysfunction, followed by vasoconstriction and abnormal blood flow due to leukocyte adhesion, which can directly induce the death of cardiac and endothelial cells (Fig. 5) [97]. Acute MI is characterized by extensive cardiomyocyte necrosis and general inflammation [98]. Increased levels of circulating mtDNA are associated with large-scale necrosis. Moreover, mtDNA content is reduced after reperfusion of the ischemic myocardium, indicating a close connection between myocardial damage and circulating mtDNA levels.

In diabetic patients, higher levels of mtDNA are positively related to the incidence of coronary artery disease, implying the potential role of mtDNA-induced inflammatory responses in the progression of coronary artery disease. Regulation of the mtDNA sensor can also modulate cardiac damage in experimental models of ischemic heart disease. Genetic depletion of *cGAS* or *STING* in mice results in the abnormal expression of IFN-downstream genes, including CXCL10 [99]. Disturbance of IRF3-dependent signaling impairs the production of inflammatory cytokines and chemokines, reduces ventricular dilation, and improves cardiac function following MI. Therefore, in MI, the cGAS-related signaling pathway is crucial for mediating the mtDNA-induced inflammatory response.

Conversely, TLR9 is not essential for mediating the mtDNA-induced inflammatory response upon cardiac ischemic injury, as revealed by *TLR9*-deleted mice [100]. Although the pathological role of the NLRP3 inflammasome has been investigated in MI models by disturbing the expression of ASC, caspase-1, and NLRP3 in mice, the exact role of the NLRP3 inflammasome remains unclear because these reports displayed varied and complicated outcomes in cardiac ischemic injury.

### 9.2 Post-ischemic remodeling

Immediately following cardiomyocyte death, the repair and maintenance of heart integrity are initiated. The functional outcomes of cardiac remodeling and infarct size are key factors in determining prognosis (Fig. 5). TLR signaling is critical for cardiac remodeling following MI [101]. Cardiac remodeling in the central scar and peri-infarct zone requires three sequential phases to determine the integrity of the heart. The inflammatory phase is the first event, characterized by enhanced cytokine production and leukocyte recruitment. The second phase is proliferation, which involves matrix protein deposition and fibroblast transdifferentiation. The final phase is maturation, which is accompanied by collagen-based scar formation. After infarction, the changed matrix content induces the recruitment of circulating cells by releasing DAMPs.

TLR2 and TLR4 participate in adverse ventricular remodeling by increasing inflammation and fibrosis in mice [102]. TLR4 activation in monocytes is closely linked to heart failure in patients with MI. Targeted deletion of *NF-κB p50* enhances cardiac dysfunction after MI [103]. Therefore, TLR signaling and its downstream NF-κB pathway are potentially involved in the enhanced adverse inflammatory response, matrix breakdown, and fibrosis processes in infarcted myocardium, contributing to maladaptive remodeling.

Additionally, macrophage activation produces more degraded products involved in myofibroblast transdifferentiation. Myofibroblasts are also involved in scar maturation and wound healing. Activation of endothelial cells by DAMPs increases angiogenesis, which is beneficial for cardiac remodeling.

**Figure 6.**
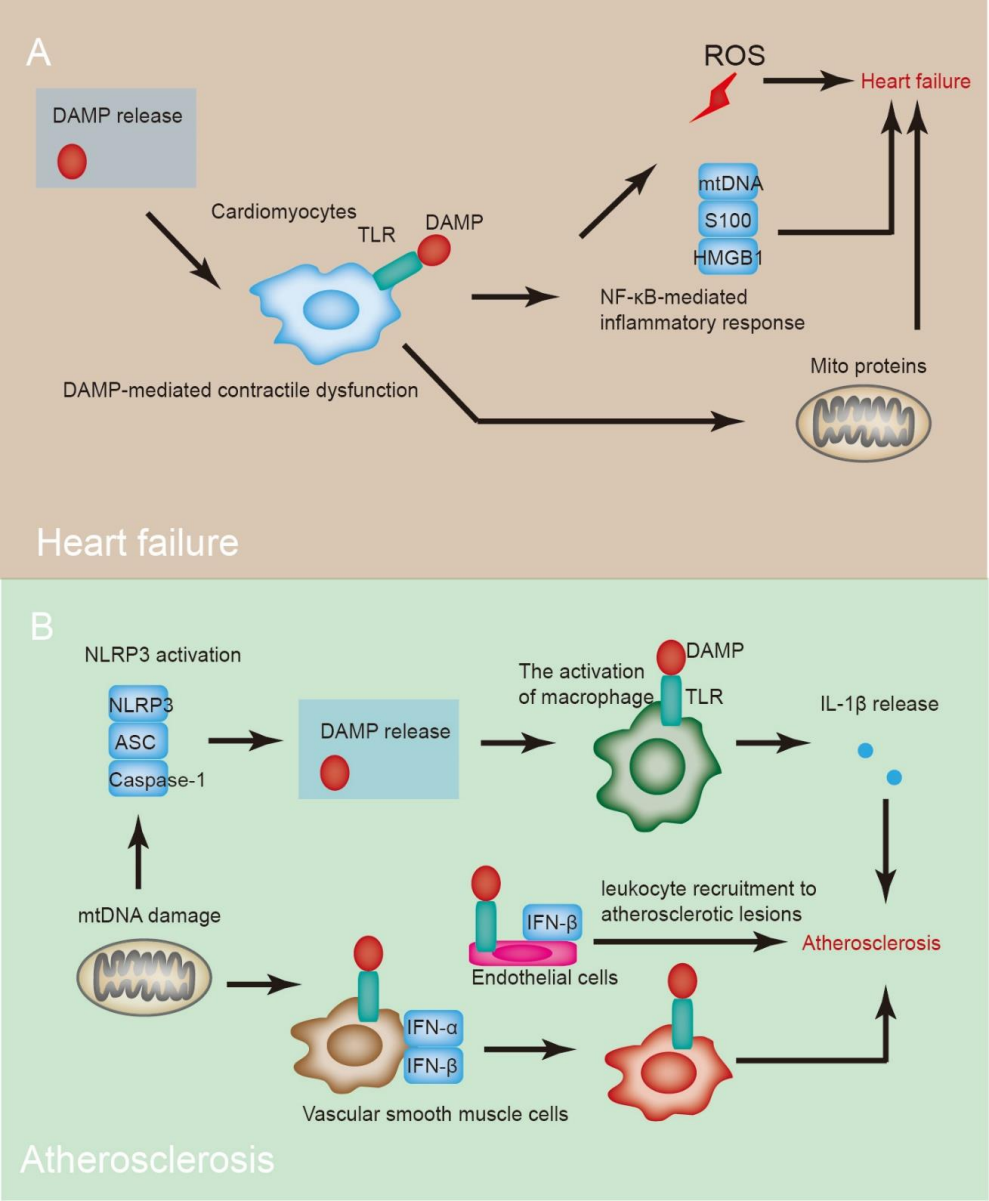
**DAMPs in heart failure and atherosclerosis**. Cardiomyocyte damage or death induces DAMP release, which further causes cardiac inflammation and triggers heart failure. Following heart failure, endogenous DAMPs, including mtDNA, intracellular S100 proteins, heat shock protein (HSP), and HMGB1, are released and recognized by TLRs to initiate an NF-κB-mediated inflammatory response. During atherogenesis, metabolites can destroy mtDNA and induce inflammation, resulting in NLRP3 inflammasome activation. NLRP3 inflammasome activation can induce IL-1β release in macrophages, which may induce atherogenesis. TLR activation induces elevated expression of IFN-α and IFN-β. IFN-α is responsible for VSMC death, and IFN-β is involved in endothelial cell adhesion and leukocyte recruitment to atherosclerotic lesions. These processes are high-risk steps in plaque formation.

### 9.3 Heart failure

Patients with heart failure present a complex interference of defective mitochondrial proteins, oxidative stress, and altered signaling pathways, leading to a dysfunctional energy supply in the myocardium. Upon heart failure, several endogenous DAMPs are released and recognized by TLRs to initiate an NF-κB-mediated inflammatory response, including mtDNA, intracellular S100 proteins, HSP, and HMGB1 (Fig. 6). In particular, mtDNA induces an inflammatory response via TLR9 in cardiomyocytes.

Heart failure can be categorized into two types: heart failure with preserved ejection fraction (HFpEF) and heart failure with reduced ejection fraction (HFrEF) [104]. Patients with HFpEF have normal systolic function but with symptoms of heart failure. Currently, the mechanism of HFpEF is probably a systemic proinflammatory condition caused by microvascular endothelial cell inflammation, encompassing concentric cardiac remodeling and diastolic dysfunction. In HFrEF, damage or death of cardiomyocytes induces DAMP release, which further causes cardiac inflammation and acts as a trigger for heart failure (Fig. 6). Currently, no direct link has been revealed between serum mtDNA levels and the severity of heart failure in patients. Heart failure presents higher serum mtDNA levels compared with healthy controls matched for age and sex [105]. mtDNA escapes from degradation and activates TLR9-mediated inflammatory responses in cardiomyocytes, myocarditis, and dilated cardiomyopathy. Accordingly, immune responses are initiated and sustained by endogenous molecules released by necrotic cells, resulting in systemic inflammation. Cardiac-specific depletion of lysosomal deoxyribonuclease (DNase) II exhibited no obvious cardiac disorders; however, it induced severe myocarditis and dilated cardiomyopathy after the induction of pressure overload. *DNase II*-deficient mice at an early stage showed inflammatory cell infiltration, increased expression of inflammatory cytokines, and accumulated mtDNA deposition in autolysosomes in the myocardium. Inhibition of TLR9, which is activated by bacterial DNA, reduces cardiomyopathy progression in *DNase II*-deficient mice. Moreover, TLR9 depletion alleviated cardiac dysfunction and inflammation induced by pressure overload, even in mice with wild-type Dnase2a alleles [106].

The roles of PRRs in heart failure have been investigated using genetic mouse models in several studies. Ablation of TLR9 in mice alleviated inflammation and cardiac injury under pressure-overload conditions [107]. This model demonstrated the association of mtDNA with cardiac inflammation, and defects in detecting mtDNA released by damaged mitochondria during mitophagy by TLR9 resulted in inefficient activation of innate immunity in heart failure. Given the role of inflammasomes in heart failure, consecutive activation of NLRP3 cannot induce an inflammatory response in the heart. Upon stimulation with LPS, transgenic mice displayed caspase-1 activation and cardiac dysfunction, whereas control mice showed no obvious cardiac injury [108]. The molecular mechanisms by which specific inflammasomes induce cardiac dysfunction require further investigation.

### 9.4 Atherosclerosis

Inflammation is a critical inducer in atherosclerosis progression [109]. During atherogenesis, metabolites, including fatty acids and cholesterol crystals, can destroy mtDNA and induce inflammation, resulting in NLRP3 inflammasome activation (Fig. 6) [110]. Activation of NLRP3 inflammasome can subsequently induce IL-1β release in macrophages, which may induce atherogenesis. IL-1α has also been proven to induce atherosclerosis, as IL-1α-ablated bone marrow transplantation into LDL receptor-null mice impairs atherosclerosis. Activation of TLR induces elevated expression of IFN-α and IFN-β. The IFN-α is responsible for vascular smooth muscle cell (VSMC) death, while IFN-β is involved in endothelial cell adhesion and recruits leukocytes to atherosclerotic lesions. These processes are high-risk steps for plaque formation (Fig. 6) [111]. Meanwhile, enhanced type 1 IFN signaling is observed in human ruptured plaques, indicating that mtDAMPs are potentially involved in atherosclerosis pathogenesis.

mtDNA damage is initiated and is correlated with plaque formation in atherosclerosis [112]. Furthermore, mtDNA damage has been correlated with plaque severity in patients. Overall, mtDAMPs are strongly associated with proatherogenic processes by producing multiple IFNs and cytokines. Inflammasome activation is also involved in this process [113]. Therefore, therapies targeting inflammatory signaling are beneficial in preventing proatherogenic processes. For instance, 8-oxoguanine glycosylase restores oxidative DNA damage, including mtDNA damage, promoting NLRP3 inflammasome activation in plaques [114]. Moreover, IRF3 signaling is essential for mediating metabolic stress-induced endothelial inflammation through the mitochondrial damage-cGAS-STING pathway. These observations verify that mtDNA and its receptors are closely associated with atherosclerosis pathogenesis. Moreover, extracellular ATP and UTP equipotentially activate mitogen-activated protein kinases via the purinergic receptor P2Y2. The mutual interaction of ATP and P2Y2 promotes the release of chemokines, such as keratinocyte-derived chemokines and macrophage inflammatory protein 2. P2Y2 deficiency lowers vascular cell adhesion molecule 1 (VCAM-1), which is known to induce leukocyte adhesion and atherosclerosis. In contrast, an enhanced ATP-P2Y2 interaction attracts leukocytes to the inflammation site, which is a crucial step in atherogenesis. Therefore, suppression of the ATP-P2Y2 axis might act as a potential target for the therapy of atherosclerotic disorders and vascular inflammation [115].

## 10.Therapeutics that alleviate cardiac inflammation

**Figure 7.**
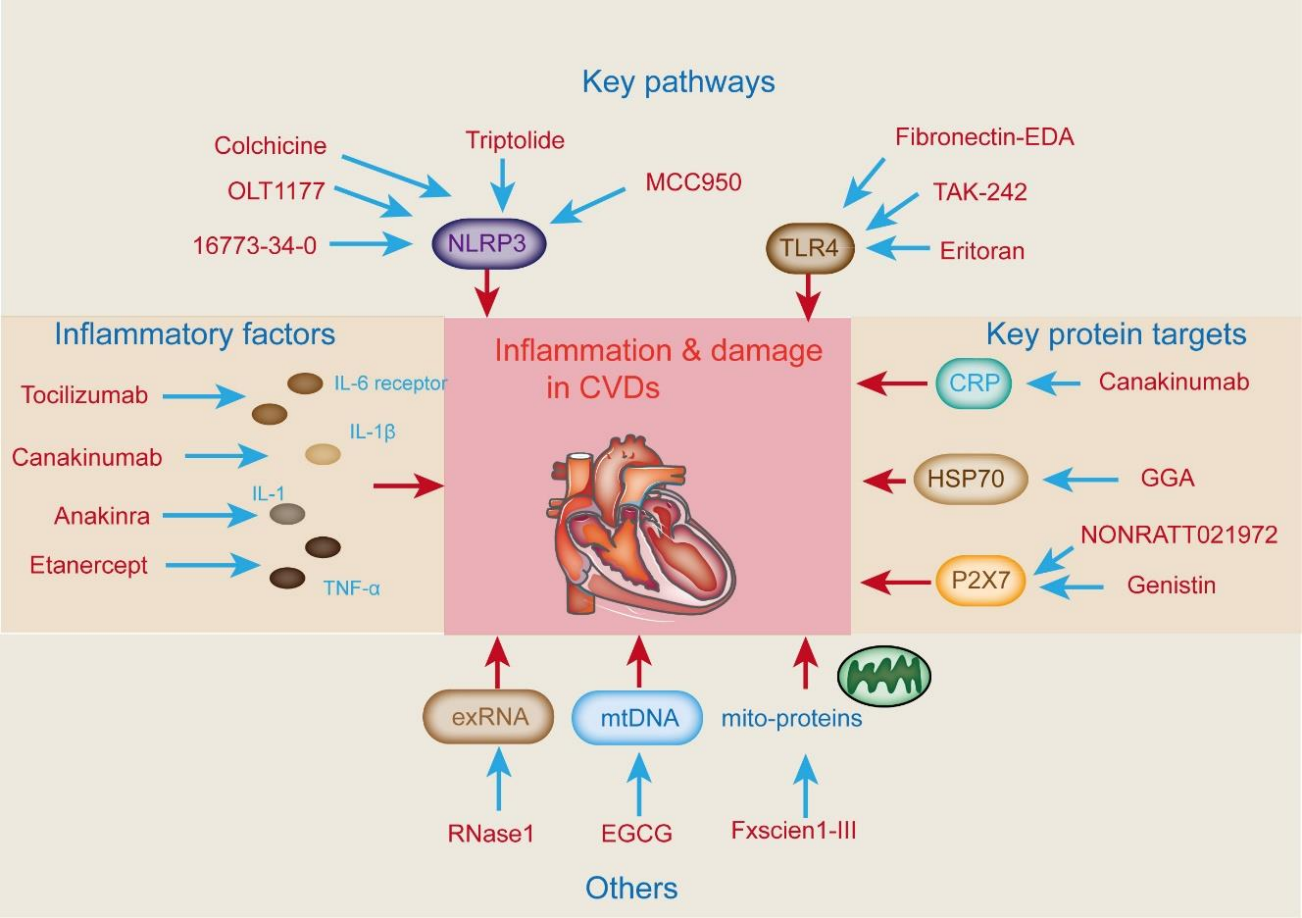
**Therapeutics targeting mtDAMPs and related signaling molecules alleviate cardiac inflammation and damage in CVDs**. The therapeutics was classified into four broad categories: key pathways, inflammatory factors, key protein targets, and others. TLR-related compounds have been designed and tested in MI and organ transplantation. Inhibition of NLRP3 inflammasome also provides a promising approach to treating CVDs. Antibodies targeting the inflammatory cytokines (tocilizumab, anakinra, etanercept, etc.) lessen cardiac inflammation and cardiac damage. Intramyocardial inhibition of P2X7, HSP70, and CRP level also acts as a potential therapeutic in CVDs. mtDNA and exRNA can be reduced by pharmacological agents and enzymes. In addition, mito-proteins, as mtDAMPs, can be therapeutic targets in CVDs.

As discussed above, immune cell-released cytokines and chemokines are highly involved in cardiac inflammation. Specifically, IL-1, IL-6, TNF-α, and IFN-γ induce cardiac inflammation. Antibodies targeting the IL-6 receptor (tocilizumab), IL-1β (canakinumab), IL-1 receptor (anakinra), and TNF-α (etanercept) reduce cardiac inflammation and damage (Fig. 7) [116]. Canakinumab reduces the recurrence of cardiovascular disorders (MI and angina) in patients with atherosclerosis and MI accompanied by decreased C-reactive protein (CRP) [117]. This protective effect is irrelevant to the lipid-lowering function, suggesting that the anti-inflammatory effect of cytokine antibodies is useful in alleviating CVDs. The effect of canakinumab on the treatment of atherosclerosis was evaluated (Fig. 7). Treatment at a dose of 150 mg every 3 months via subcutaneous injection in patients with MI attenuated the risk of recurrent cardiovascular events compared with placebo, suggesting that canakinumab potentially prevents atherosclerotic events by limiting the secretion of IL-1β by the NLRP3 inflammasome. However, these therapies may increase the risk of sepsis and fatal infections. However, administering these antibodies should be serious because they may induce infection incidence.

The secretion of inflammatory cytokines mitigates pathogen invasion. Consequently, strategies aimed at neutralizing these cytokines may impede their beneficial effects, potentially leading to increased infections. Under these circumstances, neutralization of IL-10 seems promising because IL-10 suppresses cardiac inflammation through various pathways such as SMAD, miR-375, miR-21, and p53 [118]. IL-10 also mitigates cardiac inflammation by negatively modulating IL-1, IL-6, and TNF-α levels.

Dysregulated inflammatory responses caused by mitochondrial components are believed to induce numerous human diseases, including those induced by excessive inflammatory responses and those caused by inadequate immune reactions. Bax inhibitors have been designed to provide cardioprotection in cardiovascular disorders [119]. These agents are expected to suppress mitochondrial outer membrane permeabilization (MOMP) and weaken inflammatory responses. However, recent findings suggest that pharmacological inhibition of Bax may promote mtDNA release induced by MOMP, potentially activating cGAS signaling before its cleavage and inactivation, which remains to be experimentally tested [120]. Fibronectin-EDA is upregulated upon tissue damage and may be involved in adverse cardiac remodeling following infarction [121]. Fibronectin-EDA also causes ischemic reperfusion injury (IRI) mediated by TLR4 signaling in hyperlipidemic mice. Treatment with anti-fibronectin antibody reduced infarct size following ischemia/reperfusion injury. Additionally, a clinical study observed an association between levels of fibronectin-EDA and inflammation in biopsies of heart transplantation patients with signs of chronic rejection (Fig. 7) [122]. The exact role of fibronectin-EDA in the pro-inflammatory response and as a therapeutic target following MI and heart transplantation, however, requires further investigation.

The function of HSP70 in MI is contradictory, based on published opposing results. HSP70 induces a proinflammatory response in human monocytes [123]. Nevertheless, treatment with bimoclomol promoted HSP70 levels but reduced infarct size in an MI rat model. Additionally, HSP70 induction with geranylgeranyl-acetone (GGA) showed a protective effect against MI in rats (Fig. 7) [124]. A clinical cohort study of 26 participants revealed that GGA treatment elevated HSP70 and other HSP levels in the right and left atrial appendage tissues. Additionally, GGA (400 mg/day) treatment induced upregulation of beneficial HSPs, resulting in attenuated ischemia/reperfusion injury in MI.

The inhibition of mtDNA with pharmacological agents has also been well-studied. Epigallocatechin-3-gallate (EGCG), an anti-inflammatory catechin, was previously studied in a rat model of ischemia/reperfusion injury [125]. The mtDNA levels are positively correlated with proinflammatory cytokines (TNF-α and IL-6) in the myocardium of untreated rats. Treatment with EGCE before reperfusion significantly decreased mtDNA, TNF-α, and IL-6 levels and ameliorated infarct size (Fig. 7) [126].

RNA released into the extracellular milieu (circulating extracellular RNA (exRNA)) following IRI acts as a DAMP and exerts anti-coagulatory and proinflammatory responses. RNase treatment relieved cytokine production in cardiomyocytes and attenuated infarct size in IRI mice. The protective effect of RNA inhibition was further confirmed by non-toxic RNase1 administration in a myocardial IRI mouse model as well as in the isolated IRI Langendorff-perfused rat heart, which demonstrated attenuated infarct size and preserved cardiac function [127]. A clinical study on patients with cardiac bypass surgery found that the levels of cardioprotective RNase1 were enhanced, whereas the levels of exRNA and TNF-α were decreased upon remote ischemic preconditioning by cycles of blood pressure cuff inflation around the left arm prior to the surgery. The direct effect on cardiac function and the causality between the increase in RNAse1 and a reduction in exRNA and TNF-α were not reported in this study [128]. Consequently, the exact mechanism of RNase1-induced cardioprotection remains to be elucidated.

Furthermore, the application of Exscien1-III, a fusion protein that specifically targets mitochondria and includes endonuclease III, reduced cardiac remodeling and preserved cardiac function in mice with MI [126]. Recently, mtDNA-induced cardiac injury during ischemia-reperfusion injury was due to mtDNA and HMGB1. Following ischemia-reperfusion injury, mtDNA and HMGB1 are released, leading to detrimental effects on the cardiac system [129]. Neither HMGB1 nor mtDNA alone can induce increased infarct size, whereas combined stimulation with HMGB1 and mtDNA leads to harmful effects. Although the above studies are interesting, there are currently no existing clinical trials related to mtDNA therapy in CVDs. The potential of mtDNA as a target in CVD therapy remains unclear.

TLR-related compounds have been designed and tested in MI and organ transplantation [130]. Treatment with the TLR4 antagonist eritoran reduced infarct size in an MI mouse model, accompanied by a sharp reduction in TNF-α, IL-6, and IL-1β levels [131]. Another TLR4 antagonist, TAK-242, has been suggested to reduce infarct size when used in the form of poly-(lactic-co-glycolic acid) nanoparticles (TAK-242-NP), which significantly improves the efficiency of drug delivery to macrophages and monocytes (Fig. 7) [132]. Both drugs do not reach the strict standards of clinical endpoints, and circulating immune cytokines remain unaffected.

Inhibition of NLRP3 ATPase activity with INF4E significantly reduced infarct size and improved post-ischemic left ventricular pressure in rats [133]. The NLRP3 inflammasome inhibitor 16673-34-0 decreased caspase 1 activation in cardiomyocytes stimulated with LPS and ATP. In an MI mouse model, 16673-34-0 treatment decreased infarct size (Fig. 7) [134]. Additionally, OLT1177 (dapansutrile) treatment led to a more obvious decrease in infarct size in mice (Fig. 7) [135]. The NLRP3 inflammasome-TGF-β1 axis is critical for the progression of cardiac inflammaging and fibrosis, and targeting this pathway may provide beneficial effects in cardiac inflammaging. Additionally, triptolide (a Chinese herbal compound) treatment reduced IL-18 and IL-1β cytokines and inactivated the NLRP3 inflammasome-TGF-β1 axis (Fig. 7) [136]. Furthermore, MCC950 (a specific inhibitor of the NLRP3 inflammasome) prevents heart failure by suppressing NLRP3 inflammasome and IL-1β release. Colchicine can target NLRP3 in atherosclerosis and is used to treat gouty arthritis, familial Mediterranean fever, and pericarditis. Colchicine treatment in patients with acute coronary syndrome reduces the production of IL-1β and IL-18, mediated by the NLRP3 inflammasome [137]. Short-term administration of colchicine limited infarct size in patients with ST-segment elevation myocardial infarction treated with primary percutaneous coronary intervention [138]. Another study revealed that patients with acute coronary syndrome who underwent low-dose oral colchicine and standard medical therapy during hospitalization and continued for 12 months showed no significant difference in the rate of the primary composite outcome of death, acute coronary syndromes, ischemia-driven urgent revascularization, and stroke compared with standard medical therapy alone. Colchicine group presented a higher total mortality [139].

**Figure 8.**
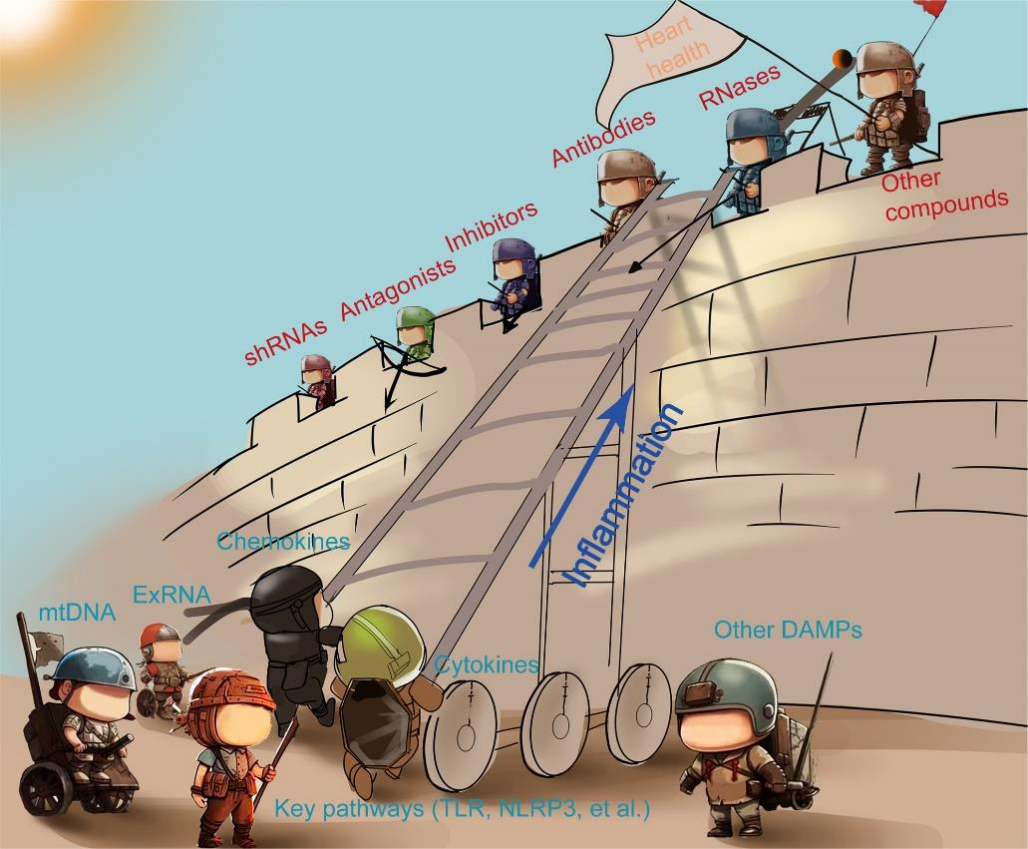
**Multiple treatment strategies protect heart health by warding off inflammation caused by DAMPs**. A variety of treatment strategies protect heart health by blocking inflammation, such as antibodies (tocilizumab, et al.), inhibitors (Bax inhibitors, et al.), antagonists (Eritoran, et al), shRNAs (P2X7 shRNA, et al.), RNases (RNase I, et al.), and other compounds. The inflammatory factors including chemokines, cytokines, mtDNA, exRNA, several key pathways (TLR, NLRP3, et al.) and other DAMPs.

Intramyocardial inhibition of P2X7 expression by short hairpin RNA (shRNA) injection following MI induction led to P2X7 signaling inhibition, reduced infiltration of circulating cells, and subsequently reduced infarct size [140]. The beneficial effect is also associated with the inhibition of the AKT and ERK1/2 pathways and the activation of the NF-κB pathway. Another lncRNA-related study found that NONRATT021972 inhibited P2X7 expression and improved cardiac function in an MI rat model (Fig. 7). Moreover, genistin reduced P2X7 levels in myocardial tissues and the levels of proinflammatory cytokines in the serum (Fig. 7). In summary, if heart health is like a city, then a variety of different treatment strategies, such as antibodies, inhibitors, antagonists, etc., are the guardians of the city, working together to defend against the inflammatory attack caused by DAMPs (Fig. 8).

## 11.Outlook

DAMPs, a class of substances released into the intercellular space or blood circulation after damage or necrosis of the body, can be used as danger signals through TLR, RIG-I-like receptors, NOD-like receptors, and other pattern recognition receptors, which stimulate the immune system and play a vital role in the occurrence and development of arthritis, atherosclerosis, thrombosis, MI, cerebral infarction, and other related diseases. The relevant DAMPs discovered in contemporary immunology mainly include HMGB1, belonging to the protein family, and ATP and uric acid, belonging to the small-molecule compound family, with a very limited number. Therefore, discovering a new type of DAMP family and deepening the understanding of the molecular mechanisms by which the danger signal DAMP stimulates the immune system and triggers various related diseases are core challenges in basic immunology. If mtDAMPs can be measured and quantified as biomarkers, they can be used to predict the response and prognosis of disease treatment.

Several mitochondrial components and metabolites can act as DAMPs that promote inflammation when released into cellular solutes or extracellular environments, and uncontrolled inflammatory responses can exacerbate CVD progression. Consequently, targeting these products improves DAMPs and the resulting CVDs. In pre-clinical models, navumab, ipiximab, and pembrolizumab increase myocardial immune infiltration and vascular inflammation. All these inhibitors increase DAMPs, NLRP3/IL-1β in myocardial tissues, and MyD88 expression.

Above all, we discussed the role of mtDAMPs in disease models and CVDs. Although the critical roles of mtDAMPs in CVDs are supported by ample data, several questions remain unresolved. First, how mtDAMPs interact with each other to initiate the immune response in vivo remains unclear. For instance, multiple mtDAMPs (TFAM and NFP) synergistically stimulate immune responses. Each mtDAMP likely exerts additional biological functions when interacting with each other. Second, it remains intriguing which type of cell death is responsible for releasing DAMPs in patients, as some mtDAMPs can be released due to multiple types of cell death. ATP is released by necrotic and apoptotic cells. During CVD progression, multiple types of cell death occur in various cell types and tissues. Third, the specific cell type that releases mtDAMPs and initiates the immune response remains a challenge. Although extracellular ATP stimulates the immune response in lung diseases, circulating ATP secreted from red blood cells has beneficial consequences in pulmonary hypertension, suggesting the diverse roles of mtDAMPs secreted from different cell types in various pathological contexts.

Extensive studies have identified multiple mtDAMPs that act on diverse PRRs. Mutual interactions are believed to induce an inflammatory response, resulting in harmful outcomes in cardiovascular diseases. These signaling molecules have been used as potential therapeutic targets for several decades. However, clinical translation of the road is not straightforward. Clinical trial outcomes targeting mtDAMPs can vary due to the monocentric and unstandardized study design. Future clinical studies targeting mtDAMPs/PRRs should be conducted in multi-centric, standardized, and pre-conducted in clinically similar animal models. In addition, the reproducibility of clinical studies is important for different locations and scenarios. Translational research should also focus on deciphering the complex interactions and cross-talk between these mediators. Finally, the mechanistic pathways of these mediators in cardiovascular diseases in human studies are also warranted to determine whether they have mechanisms similar to those in animal models. Considering the vital effect of mtDAMPs on CVDs and the positive correlation between mtDAMP levels and disease severity, it is valuable to decipher therapeutic insights into modulating mtDAMPs. Modulation of the release and signaling pathways of mtDAMPs may offer beneficial effects in relieving the inflammatory response and pathogenesis of CVDs.
